# Supplementary material for: Keras/TensorFlow in Drug Design for Immunity Disorders
Source: Int J Mol Sci. 2023 Oct 9;24(19):15009. doi: 10.3390/ijms241915009 (PMC10573944; doi:10.3390/ijms241915009)
Supplement: Supplementary file 1 [file ijms-24-15009-s001.zip › ijms-2628860-supplementary.pdf]

Supplementary S1

Keras/TensorFlow in Drug Design for Immunity Disorders

Paulina Dragan[1], Kavita Joshi[1], Alessandro Atzei[1, 2], Dorota Latek[1, *]

[1]Faculty of Chemistry, University of Warsaw, Pasteura 1, 02-903 Warsaw, Poland

[2]Department of Life and Environmental Science, Food Toxicology Unit, University of Cagliari, University Campus of Monserrato, SS 554, 09042 Cagliari, Italy.

*Correspondence: dlatek@chem.uw.edu.pl

Figure S1. The sequence alignment of the templates (top: 5LWE, middle: 6MEO) and CXCR3-a used as the input for multiple template modeling in Modeller. The microswitches described in Fig. 1. have been highlighted for CXCR3: the W6.48 toggle switch with F6.44 from the PIF motif—blue, the Y7.53 toggle switch—green, and the R3.50 ionic lock with Y4.58 interacting with it in the active state—orange.

```
5lwe          ------------------------------------------------------------   0
6meo          --------------------------MDYQVSSPIDINYTSEPCQKINVKQIAARLLPPL   34
cxcr3-a       MVLEVSDHQVLNDAEVAALLENFSSSYDYGE-NESDSCCTSPPCPQDFSLNFDRAFLPAL   59


5lwe          YWLVFIVGALGNSLVILVYWYCARAKTATDMFLLNLAIADLLFLVTLPFWAI--------   52
6meo          YSLVFIFGFVGNMLVILILINCKRLKSMTDIYLLNLAISDLFFLLTVPFWAHYAAAQWDF   94
cxcr3-a       YSLLFLLGLLGNGAVAAVLLSRRTALSSTDTFLLHLAVADTLLVLTLPLWAVDAAVQWVF   119
              * *:*:.* :**  *  :         : ** :**:**::* ::::*:*:**

5lwe          ATFMCKVVNSMYKMNFYSCVLLIMCICVDRYIAIAQAMRAHTWREKRLLYSKMVCFTIWV   112
6meo          GNTMCQLLTGLYFIGFFSGIFFIILLTIDRYLAVVHAVFAL--KARTVTFGVVTSVITWV   152
cxcr3-a       GSGLCKVAGALFNINFYAGALLLACISFDRYLNIVHATQ--LYRRGPPARVTLTCLAVWG   177
              .. :*::  .:: :.*::  :::  : .***: :.:*       :        :...  *

5lwe          LAAALCIPEILYCTTKLK-------------------SAVLALKVILGFFLPFVVMACCY   153
6meo          VAVFASLPGIIFTRSQKEGLHYTCSSHFPYSQYQFWKNFQTLKIVILGLVLPLLVMVICY   212
cxcr3-a       LCLLFALPDFIFLSAHHDERLNAT--HCQYNFPQVGRTALRVLQLVAGFLLPLLVMAYCY   235
              :.   .:* :::  :: .                .      :: *:.**::**. **

5lwe          TIIIHTLIQAKK-SSKHKALKATITVLTVFVLSQFPYNCILLVQTIDAYAMFISNCAVST   212
6meo          SGILKTLLRCRNEKKRHRAVRLIFTIMIVYFLFWAPYNIVLLLNTFQEFFG-LNNCSSSN   271
cxcr3-a       AHILAVLLVSRG-QRRLRAMRLVVVVVVAFALCWTPYHLVVLVDILMDLGALARNCGRES   294
              : *: .*: .:   . : :*::   ..:: .: *    **: ::*:: :        **. ..

5lwe          AIDICFQVTQAIAFFHSCLNPVLYVFVGERFRRDLVKTLKNLGAISQAAAHH--------   264
6meo          RLDQAMQVTETLGMTHCCINPIIYAFVGEKFRNYLLVFFQ--------------------   311
cxcr3-a       RVDVAKSVTSGLGYMHCCLNPLLYAFVGVKFRERMWMLLLRLGCPNQRGLQRQPSSSRRD   354
               :* . .**. :.  *.*:**::*.*** :**. :   :

5lwe          --------------       264
6meo          --------------       311
cxcr3-a       SSWSETSEASYSGL       368
```

Figure S2. Multiple sequence alignment of chemokine receptors CCR2, CCR3, and CXCR3. The most conserved residues are highlighted in yellow and used for residue numbering in the Ballesteros-Weinstein notation [1]. The alignment was performed using Clustal Omega [2].

```
CLUSTAL O(1.2.4) multiple sequence alignment


sp|P49682|CXCR3_HUMAN      MVLEVSDHQVLNDAEVAALLENFSSSYDYGENESDSCCTSPPCPQDFSLNFDRAFLPALY     60
sp|P41597|CCR2_HUMAN       -MLSTSRSRFIRNTNESGEEVTTFFDYDY----------GAPCHKFDVKQIGAQLLPPLY     49
sp|P51677|CCR3_HUMAN       ---------MTTSLDTVETFGTTSYYDDV----------GLLCEKADTRALMAQFVPPLY     41
                                    .  . :      .     *           .  * :     :   ::* **
                                    1.50                        2.50
sp|P49682|CXCR3_HUMAN      SLLFLLGLLGNGAVAAVLLSRRTALSSTDTFLLHLAVADTLLVLTLPLWAVDA-AVQWVF     119
sp|P41597|CCR2_HUMAN       SLVFIFGFVGNMLVVLILINCKKLKCLTDIYLLNLAISDLLFLITLPLWAHSA-ANEWVF     108
sp|P51677|CCR3_HUMAN       SLVFTVGLLGNVVVVMILIKYRRLRIMTNIYLLNLAISDLLFLVTLPFWIHYVRGHNWVF     101
                           **:* .*::**  *. :*:. :     *: :**:**::* *:::***:*   . . :***
                                                       3.50                       4.50
sp|P49682|CXCR3_HUMAN      GSGLCKVAGALFNINFYAGALLLACISFDRYLNIVHATQLYRRGPPARVTLTCLAVWGLC     179
sp|P41597|CCR2_HUMAN       GNAMCKLFTGLYHIGYFGGIFFIILLTIDRYLAIVHAVFALKARTVTFGVVTSVITWLVA     168
sp|P51677|CCR3_HUMAN       GHGMCKLLSGFYHTGLYSEIFFIILLTIDRYLAIVHAVFALRARTVTFGVITSIVTWGLA     161
                           * .:**:  .::: . :.  :::  :::**** ****.   :    :  .:*.: .* :.
                                                                                  5.50
sp|P49682|CXCR3_HUMAN      LLFALPDFIFLSAHHDERLNATHCQYNFPQVGRT----ALRVLQLVAGFLLPLLVMAYCY     235
sp|P41597|CCR2_HUMAN       VFASVPGIIFTKCQKED--SVYVCGPYFPR----GWNNFHTIMRNILGLVLPLLIMVICY     222
sp|P51677|CCR3_HUMAN       VLAALPEFIFYETEELF--EETLCSALYPEDTVYSWRHFHTLRMTIFCLVLPLLVMAICY     219
                           :: ::* :** . ..    .   *   :*.           :   :  ::****:*. **
                                                             6.50
sp|P49682|CXCR3_HUMAN      AHILAVLLVSRGQR-RLRAMRLVVVVVVAFALCWTPYHLVVLVDILMDLGALARNCGRES     294
sp|P41597|CCR2_HUMAN       SGILKTLLRCRNEKKRHRAVRVIFTIMIVYFLFWTPYNIVILLNTFQEFF-GLSNCESTS     281
sp|P51677|CCR3_HUMAN       TGIIKTLLRCPSKK-KYKAIRLIFVIMAVFFIFWTPYNVAILLSSYQSIL-FGNDCERSK     277
                           : *: .** . .:: : :*:*::..:: .: : ****::.:*:.   .:     :*   .
                                              7.50
sp|P49682|CXCR3_HUMAN      RVDVAKSVTSGLGYMHCCLNPLLYAFVGVKFRERMWMLLLR-----LGCPNQRGLQRQPS     349
sp|P41597|CCR2_HUMAN       QLDQATQVTETLGMTHCCINPIIYAFVGEKFRSLFHIALGCRIAP-LQKPVCGGPGVRPG     340
sp|P51677|CCR3_HUMAN       HLDLVMLVTEVIAYSHCCMNPVIYAFVGERFRKYLRHFFHRHLLMHLGRYIP----FLPS     333
                           ::* .  **. :.  ***:**::***** :**. :   :       *           *.

sp|P49682|CXCR3_HUMAN      SSRR----------DSSW--SETSEASYS---GL368
sp|P41597|CCR2_HUMAN       KNVKVTTQGLLDGRGKGKSIGRAPEASLQDKEGA374
sp|P51677|CCR3_HUMAN       EKLE--------RTSSVSPSTAEPELSIVF----355
                           .. .          ..        * *
```

Figure S3. Training efficiency of Keras/TensorFlow NN using ChEMBL ligand datasets for chemokine receptors – values of the loss function. Here, 80% of the ChEMBL-retrieved datasets were used as training sets.

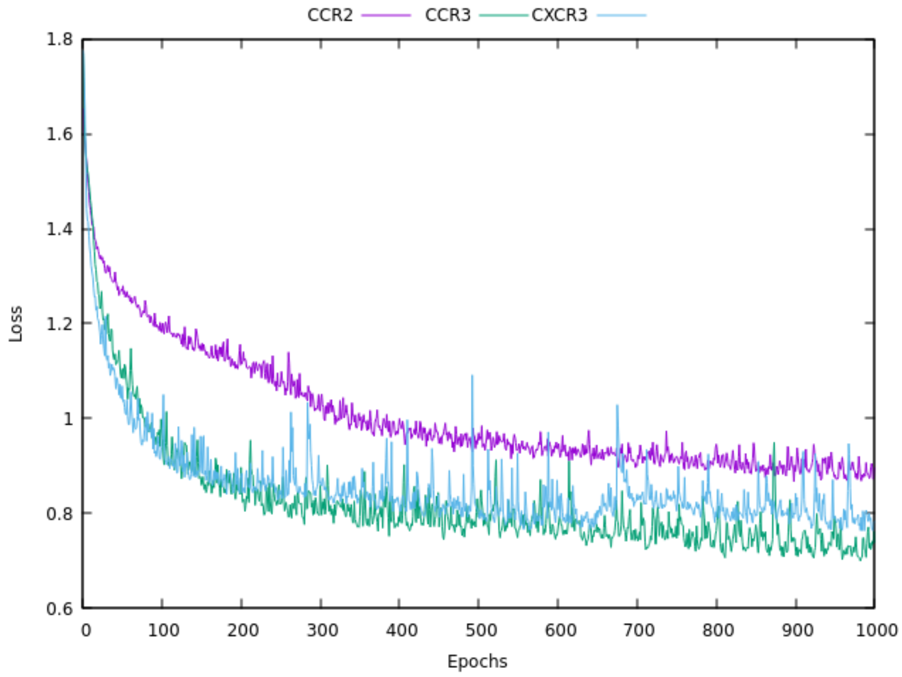

Figure S4. Training efficiency of Keras/TensorFlow NN using ChEMBL ligand datasets for chemokine receptors – the model accuracy. Here, 80% of the ChEMBL datasets were used as training sets.

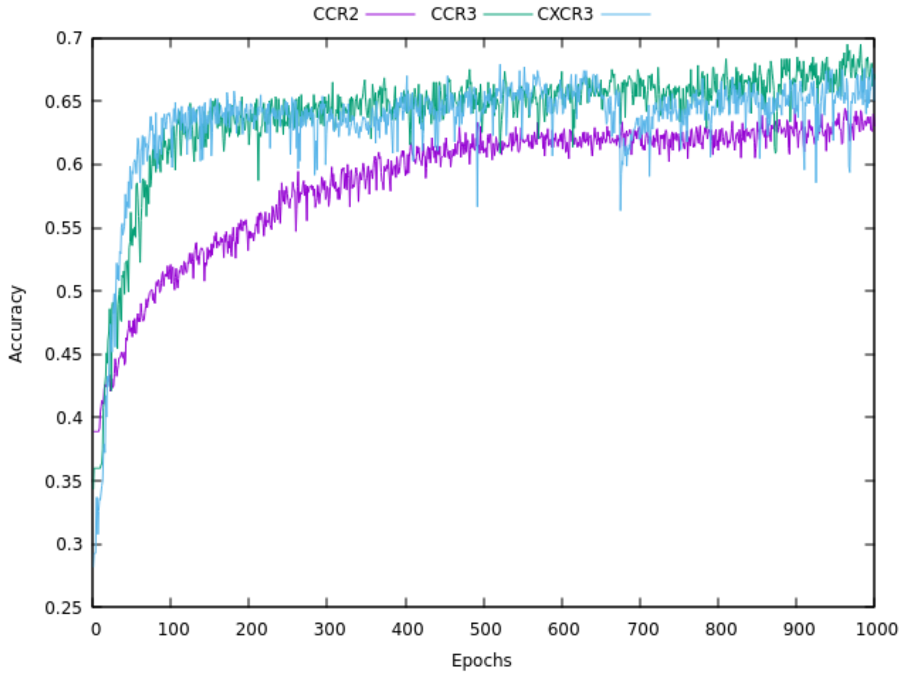

Figure S5. The impact of the quantity of the datasets on the model accuracy. Here, either 80% or 40% of the ChEMBL datasets were used as training sets.

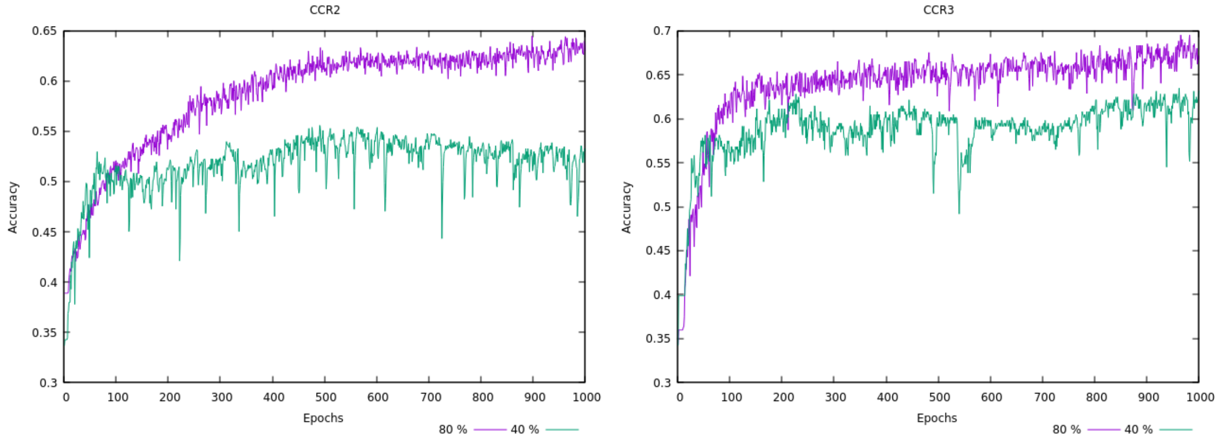

Figure S6. Training efficiency of Keras/TensorFlow NN using ChEMBL ligand datasets for the CB1 cannabinoid receptor. Here, 80% of the ChEMBL-retrieved datasets were used as training sets.

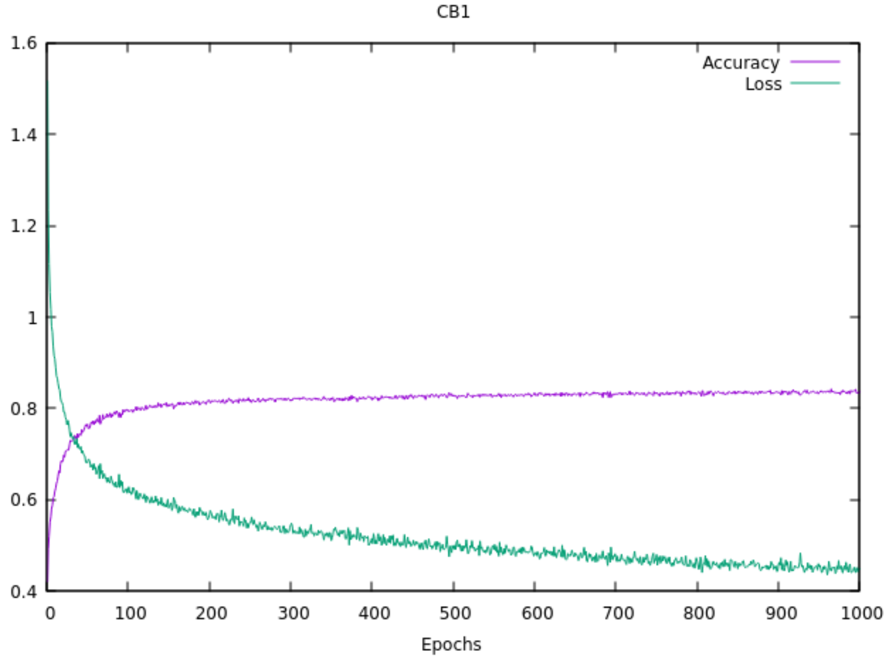

Figure S7. Training efficiency of Keras/TensorFlow NN using ChEMBL ligand datasets for the CB2 cannabinoid receptor. Here, 80% of the ChEMBL-retrieved datasets were used as training sets.

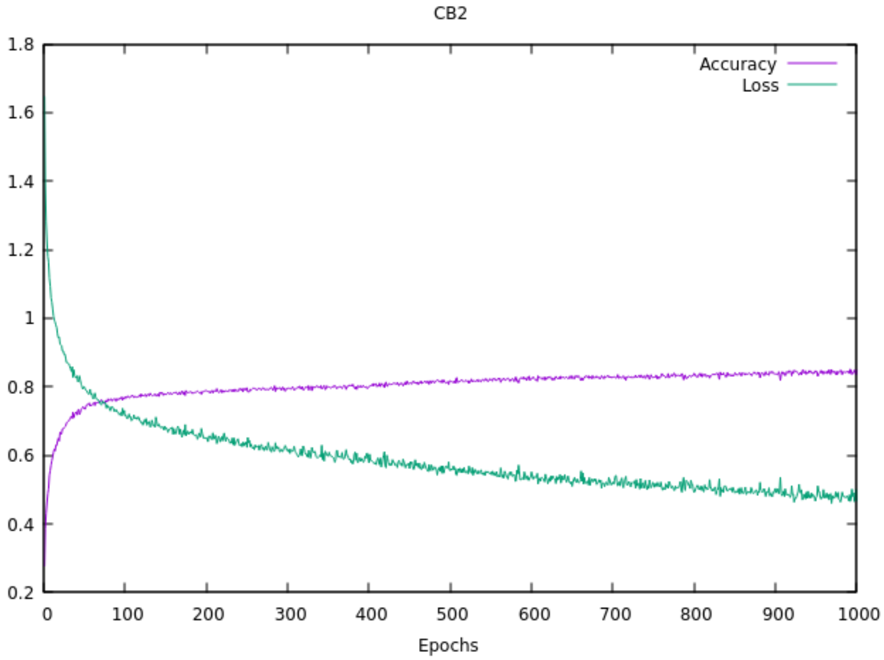

Table S1. The ligand-receptor interactions determined by Maestro [3] for the SBVS and MD results for CCR2.

Compound id in Enamine HLL	SBVS-based ligand-receptor interactions	MD-based ligand-receptor interactions
Z144527132	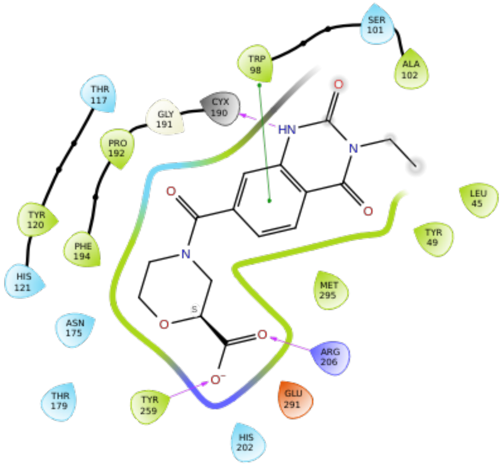	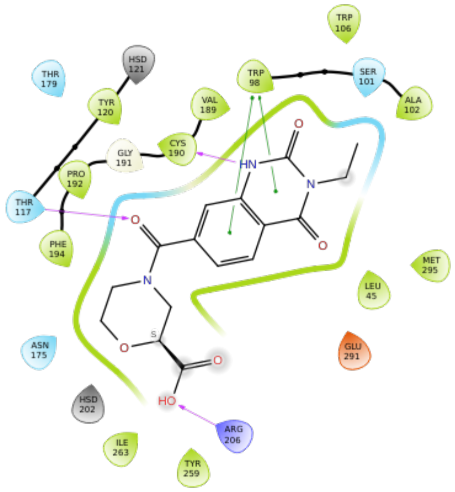
Z199951150	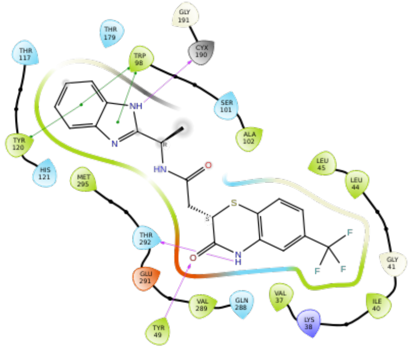	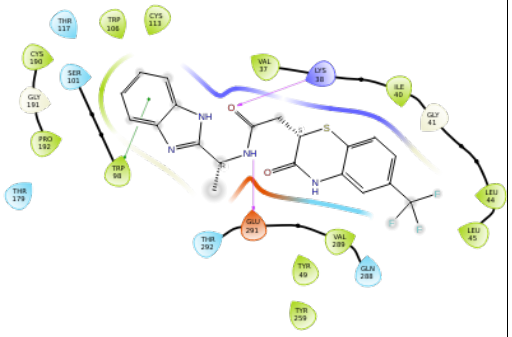

Z2607653088	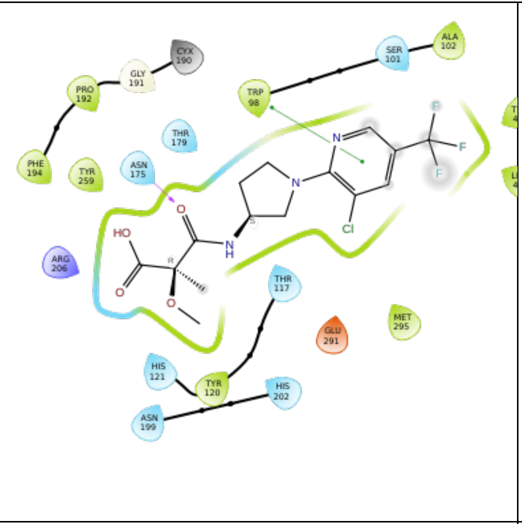	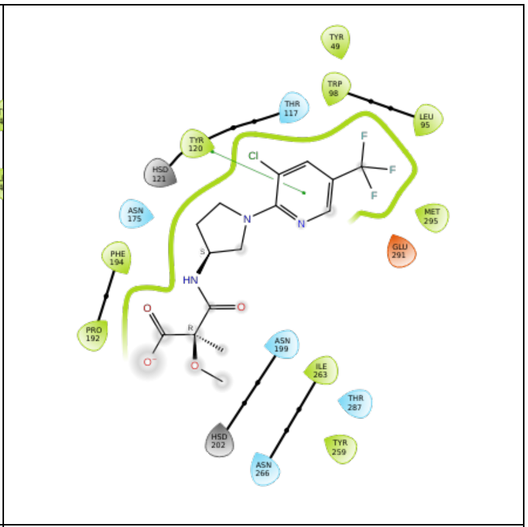
Z45637008	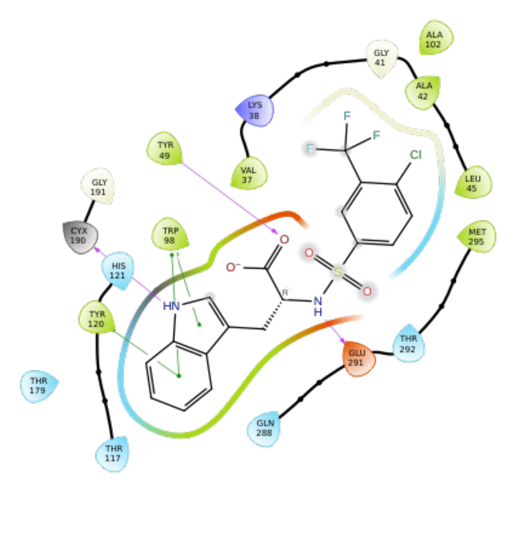	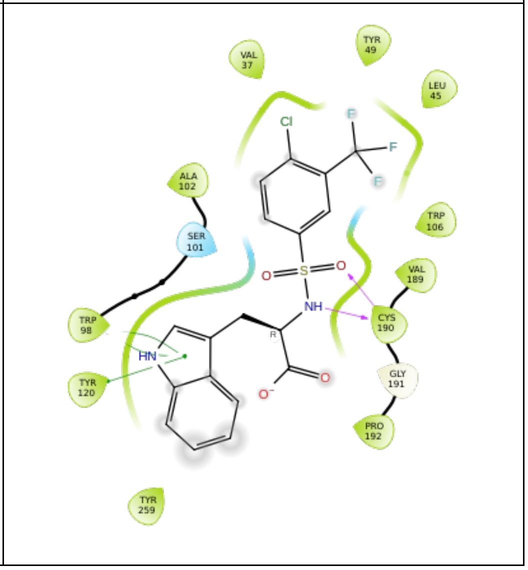

Charged (negative)
Charged (positive)
Glycine
Hydrophobic
Metal
Polar
Unspecified residue
Water
Hydration site
Hydration site (displaced)
Distance
H-bond
Halogen bond
Metal coordination
Pi-Pi stacking
Pi-cation
Salt bridge
Solvent exposure

Table S2. The ligand-receptor interactions determined by Maestro for the SBVS and MD results for CCR3.

Compound id in Enamine HLL	SBVS-based ligand-receptor interactions	MD-based ligand-receptor interactions
Z1274732994	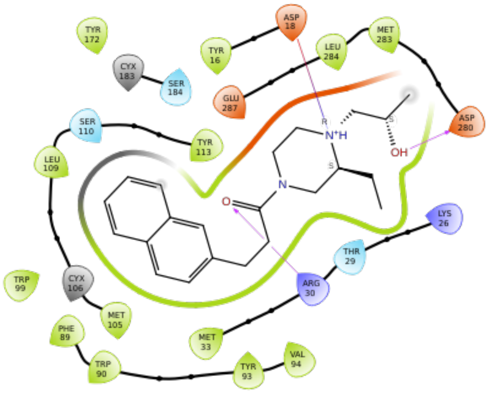	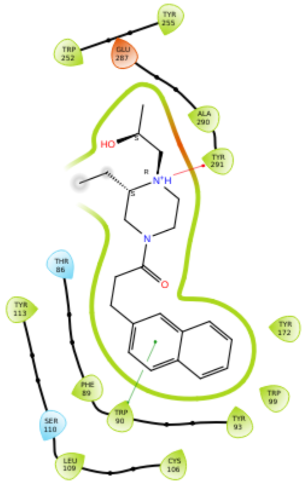
Z1912507172	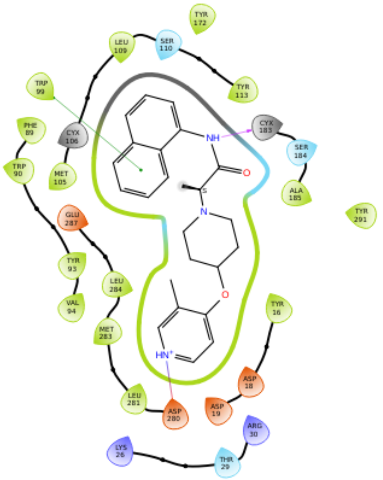	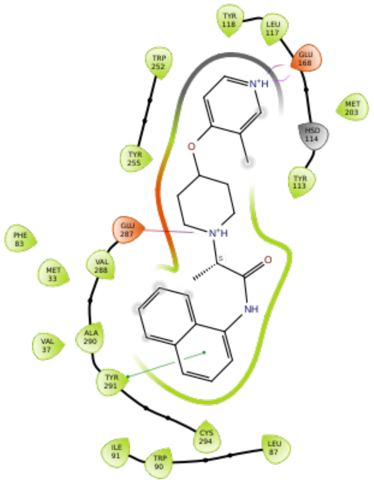

Z2441027668	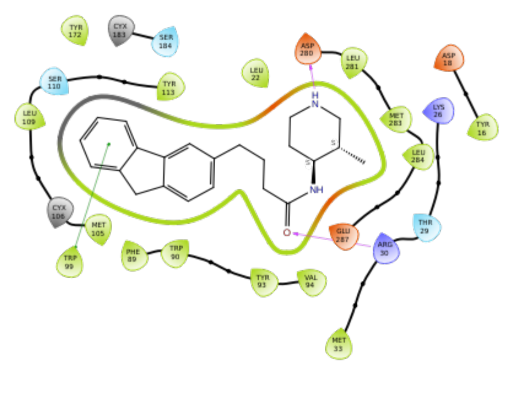	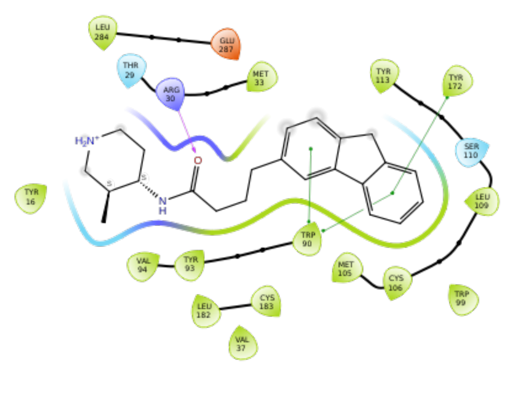
Z2606182917	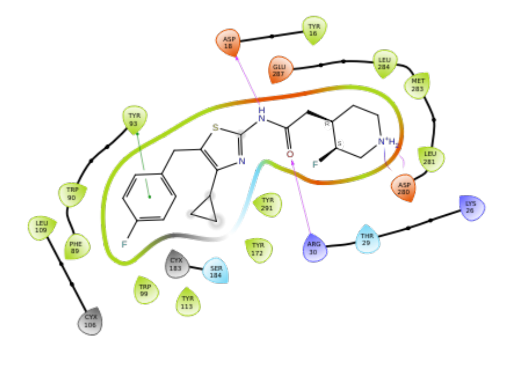	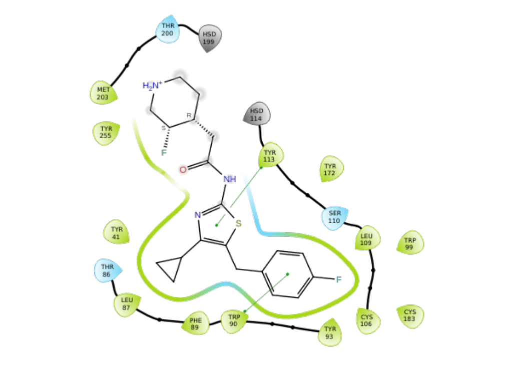
Z2764968046	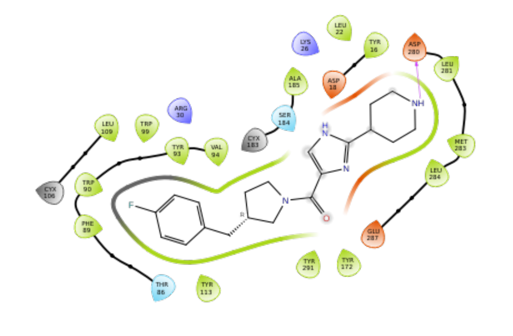	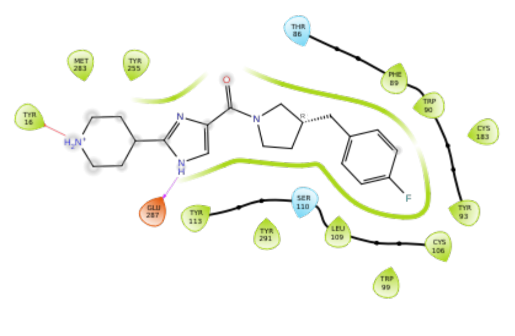

Charged (negative)
Charged (positive)
Glycine
Hydrophobic
Metal
Polar
Unspecified residue
Water
Hydration site
Hydration site (displaced)
Distance
H-bond
Halogen bond
Metal coordination
Pi-Pi stacking
Pi-cation
Salt bridge
Solvent exposure

Table S3. The ligand-receptor interactions determined by Maestro for the SBVS and MD results for CXCR3.

Compound id in Enamine HLL	SBVS-based ligand-receptor interactions	MD-based ligand-receptor interactions
Z107207944	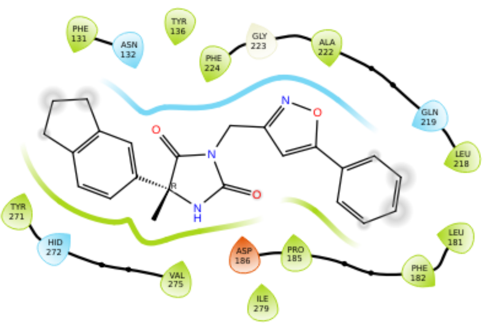	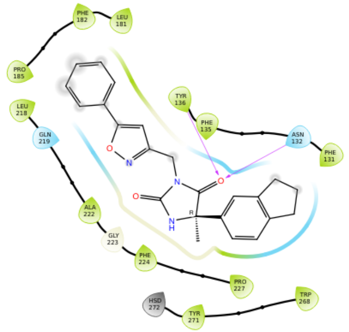
Z1167188972	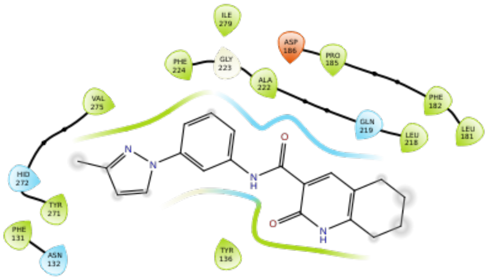	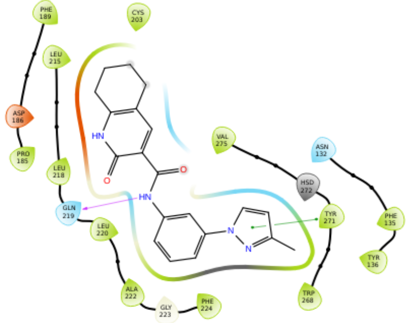
Z1510954688	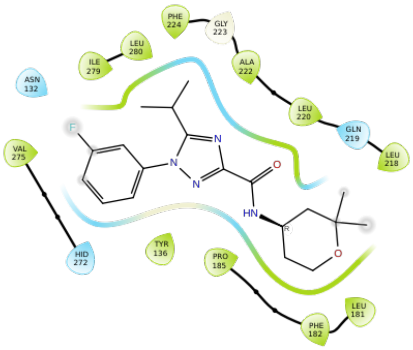	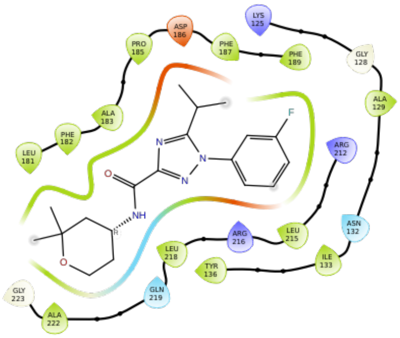

Z1903257002	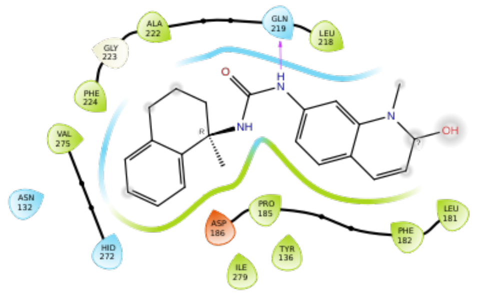	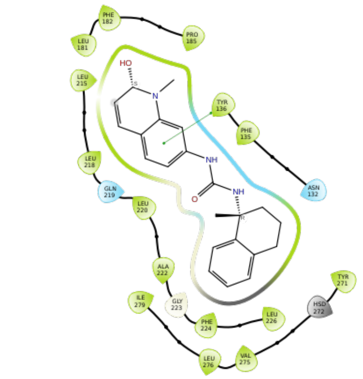
Z2233592864	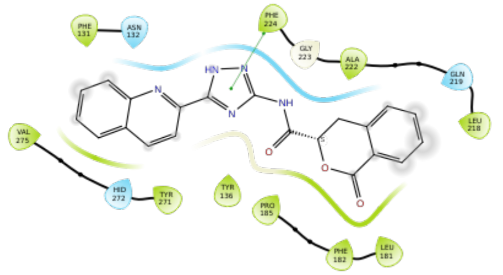	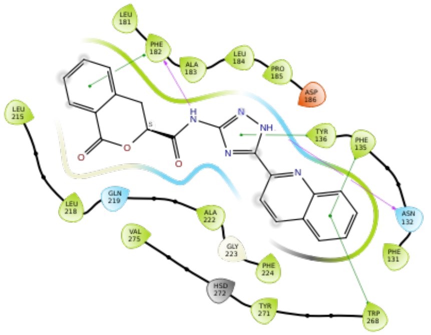

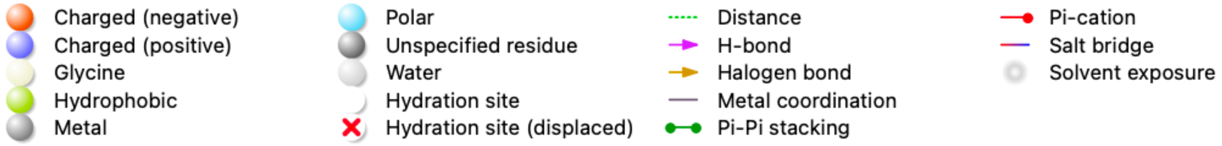

Table S4. Suggested modifications of the molecules selected for CCR2 and their contacts with the receptor predicted by Maestro. In the rightmost column: green dashed lines—good contacts, blue dashed lines—aromatic interactions, yellow dashed lines—hydrogen bonds.

Original HLL compound	Modified structure of new compound	New interactions formed with the receptor	AutoDock Vina score
Z144527132	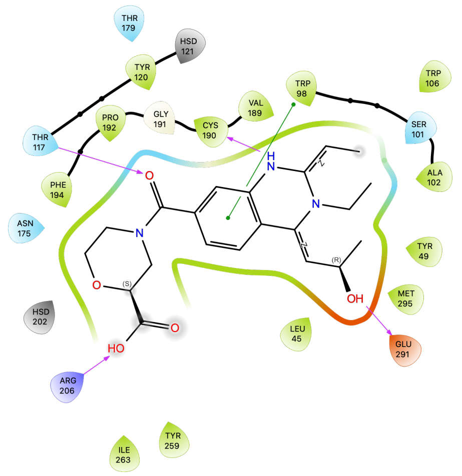	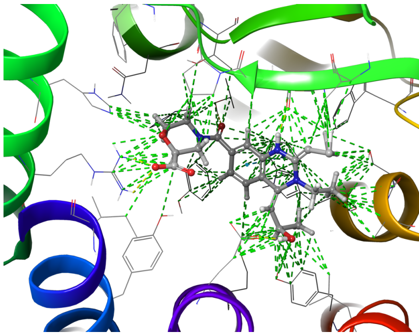	-8.508
Z199951150	—	—	-9.305
Z2607653068	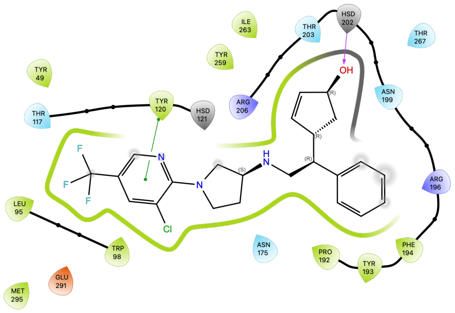	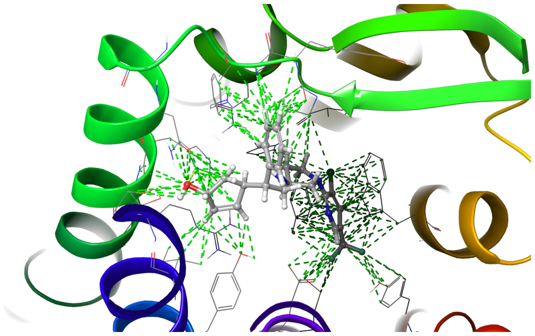	-8.339
Z45637008	—	—	-8.103

Charged (negative)
Charged (positive)
Glycine
Hydrophobic
Metal
Polar
Unspecified residue
Water
Hydration site
Hydration site (displaced)
Distance
H-bond
Halogen bond
Metal coordination
Pi-Pi stacking
Pi-cation
Salt bridge
Solvent exposure

Table S5. Suggested modifications of the molecules selected for CCR3 and their contacts with the receptor predicted by Maestro. In the rightmost column: green dashed lines—good contacts, blue dashed lines—aromatic interactions, yellow dashed lines—hydrogen bonds, purple dashed lines—salt bridges, dark green—pi-cation interactions.

Original HLL compound	Modified structure of new compound	New interactions formed with the receptor	AutoDock Vina score
Z1274732994	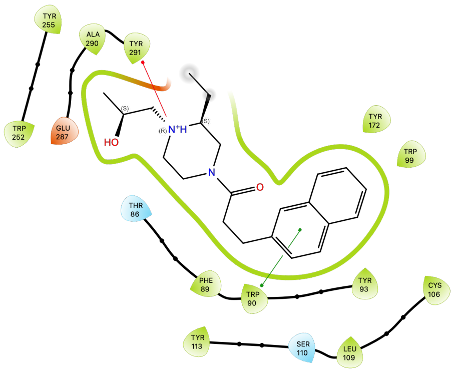	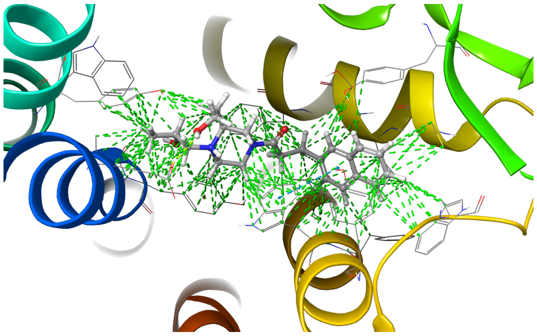	-9.796
Z1912507172	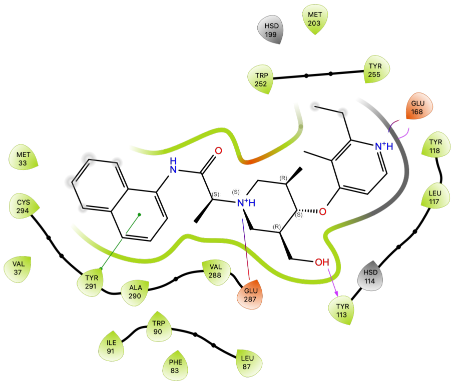	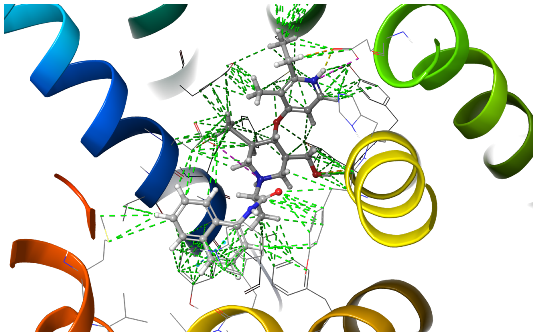	-8.995

Z2441027668	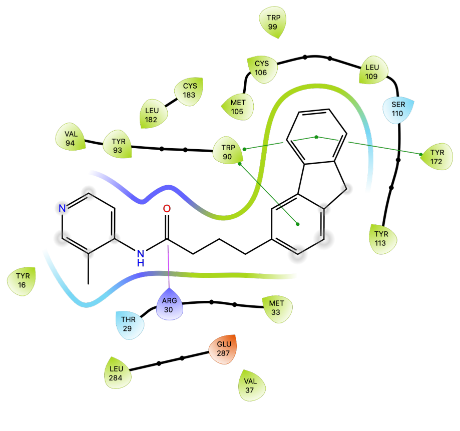	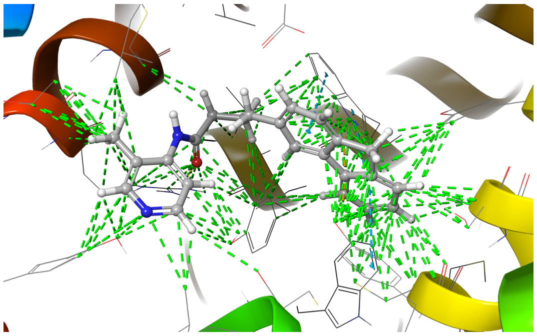	-7.486
Z2606182917	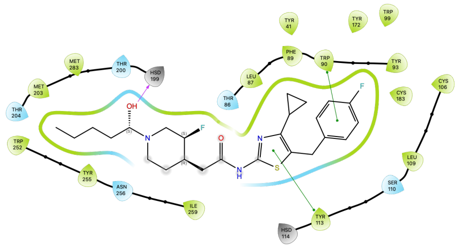	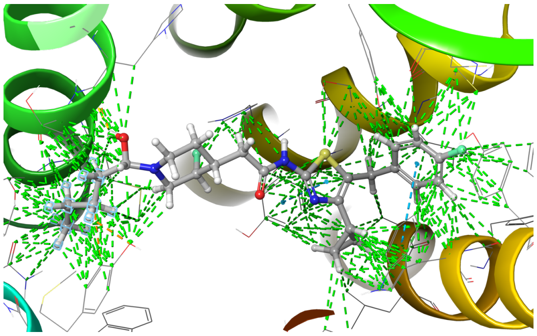	-8.387
Z2764968046	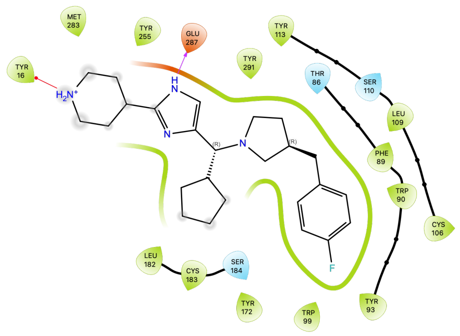	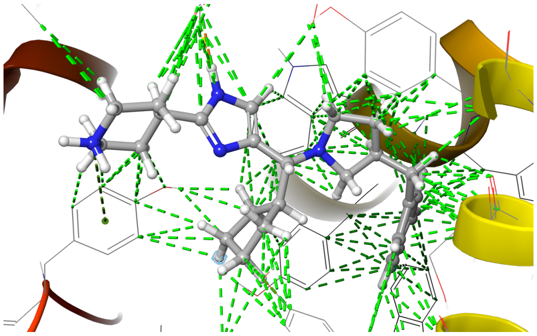	-8.926

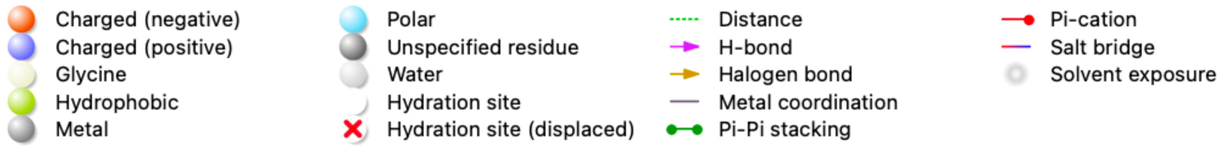

Table S6. Suggested modifications of the molecules selected for CXCR3 and their contacts with the receptor predicted by Maestro. In the rightmost column: green dashed lines—good contacts, blue dashed lines—aromatic interactions, yellow dashed lines—hydrogen bonds.

Original HLL compound	Modified structure of new compound	New interactions formed with the receptor	AutoDock Vina score
Z107207944	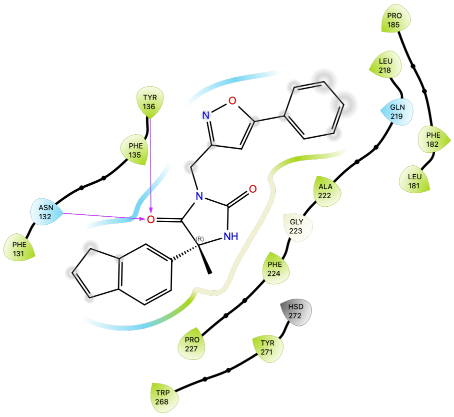	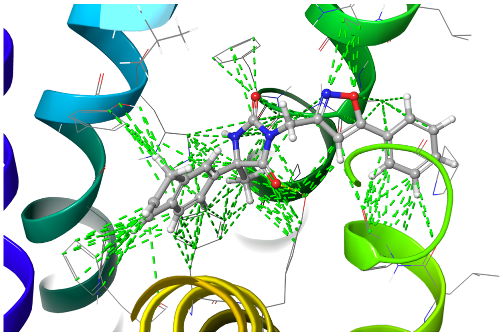	-9.558
Z1167188972	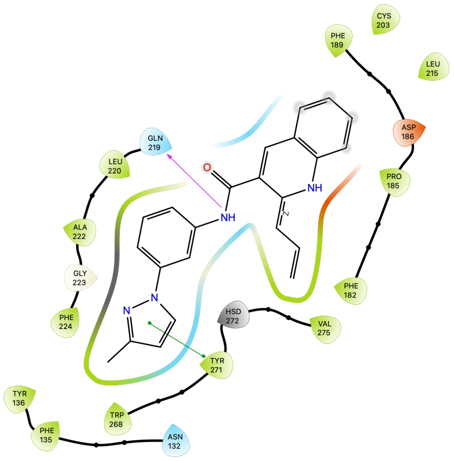	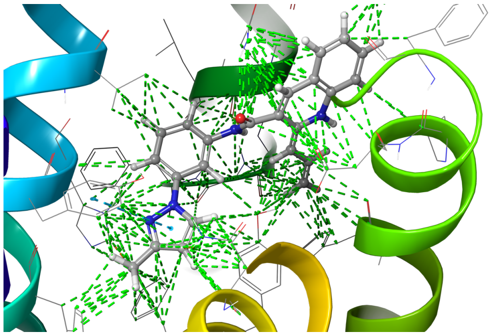	-10.688

Z1510954688	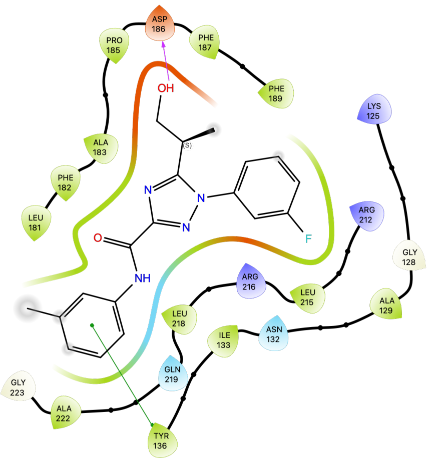	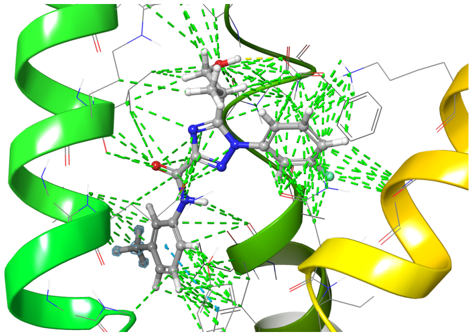	-8.422
Z1903257002	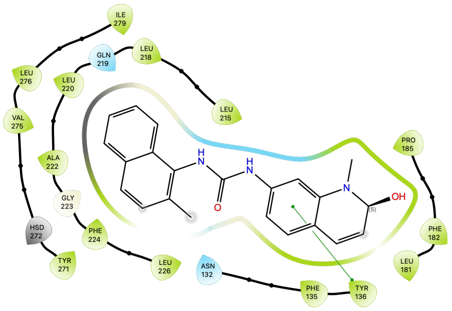	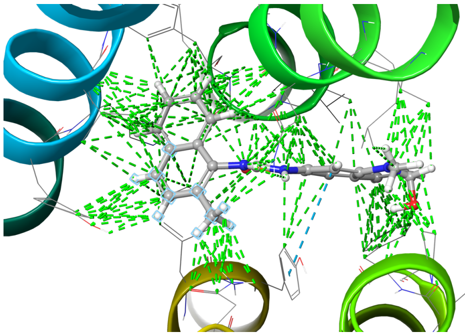	-10.318
Z2233592864	—	—	-11.862

Charged (negative)
Charged (positive)
Glycine
Hydrophobic
Metal
Polar
Unspecified residue
Water
Hydration site
Hydration site (displaced)
Distance
H-bond
Halogen bond
Metal coordination
Pi-Pi stacking
Pi-cation
Salt bridge
Solvent exposure

Table S7. Binding modes obtained from AutoDock Vina for known CXCR3 antagonists. Polar contacts have been marked with yellow dashed lines with the respective receptor residues shown in sticks. Receptor structures were shown in the blue-to-red color scheme, while ligands were shown in blue.

Known CXCR3 antagonist	AutoDock Vina prediction of its binding mode
ACT-672125	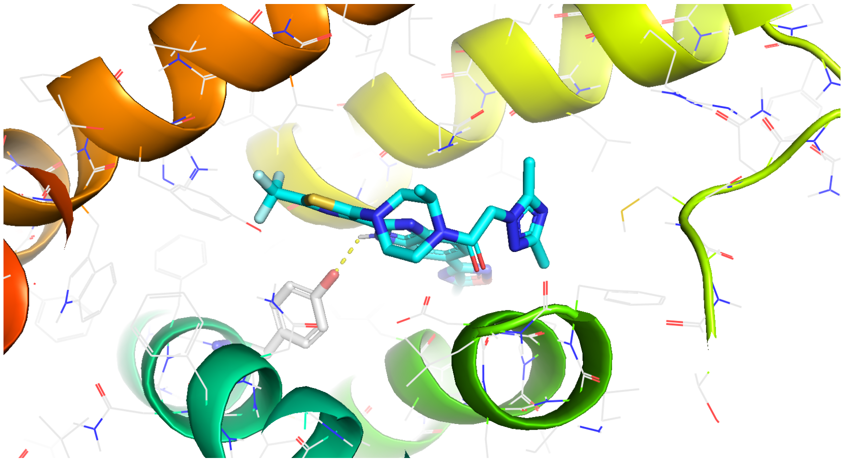
ACT-660602	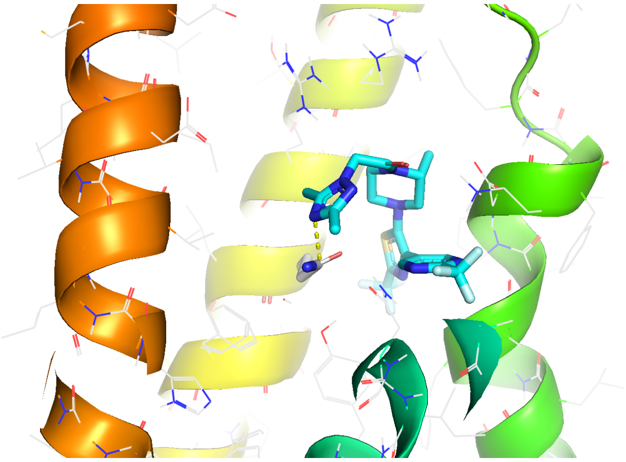

ACT-777991	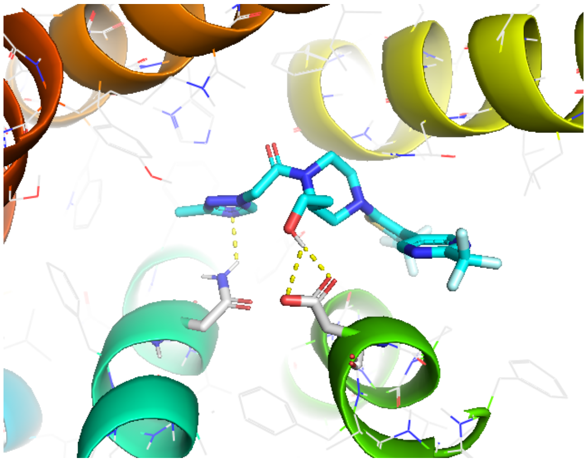
VUF10661	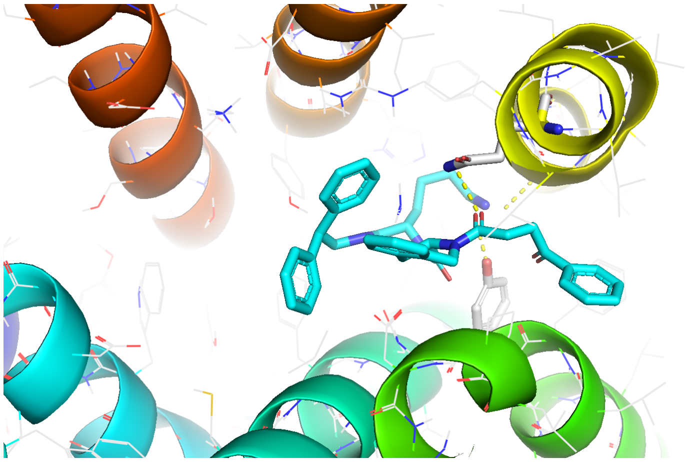

Table S8. Histograms showing the dataset classes distribution for the current and the previous CB1 and CB2 datasets (full datasets). PChEMBL values and corresponding classes used in training of NNs: 0–4 (class I), 4–5 (class II), 5–6 (class III), 6–7 (class IV), 7–8 (class V), 8–9 (class VI), above 9 (class VII).

Receptor	The current dataset	The previous dataset from https://db-gpcr.chem.uw.edu.pl
CB1	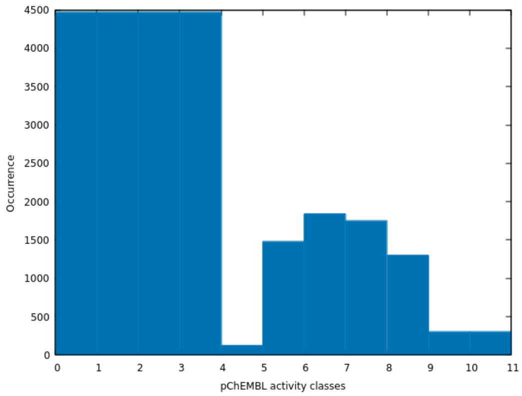	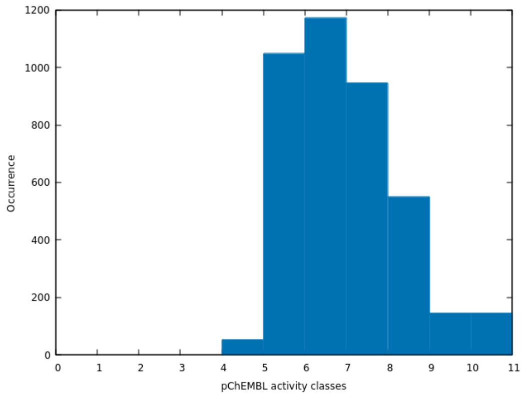
	Total of 5636 compounds	Total of 1958 compounds
CB2	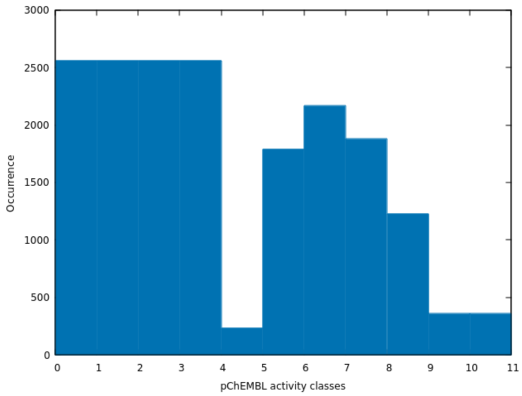	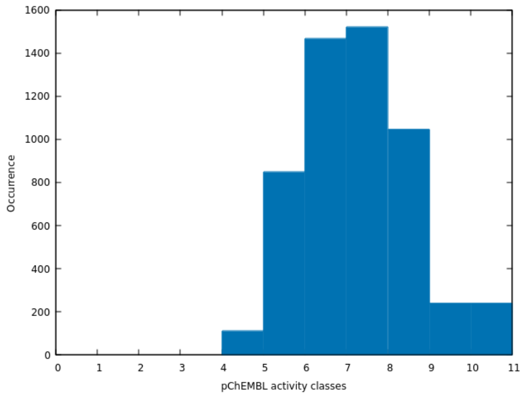
	Total of 5109 compounds	Total of 2616 compounds

Table S9. Histograms of values of Tanimoto index between the training and validation datasets.

Training set	Number of datapoints	Validation set	Number of datapoints	Histogram of values of Tanimoto index<br><br>training vs. validation set
CCR2	1995	CCR2	399	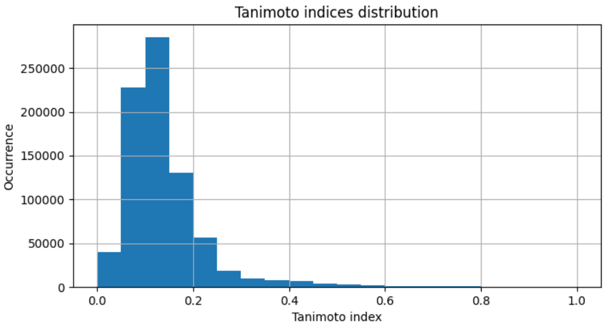
		CCR3	121	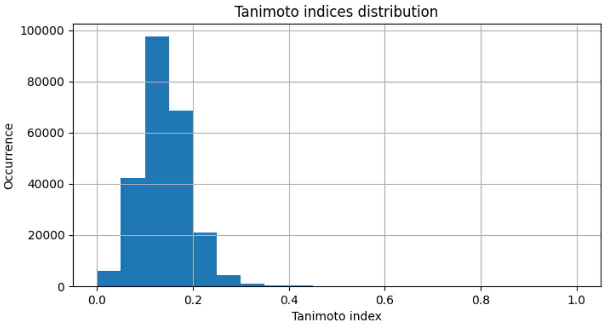
		CXCR3	199	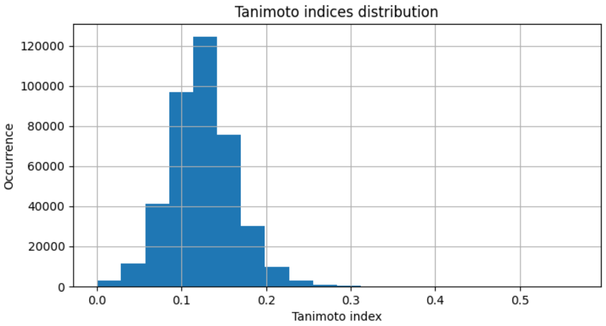
CCR3	603	CCR3	121	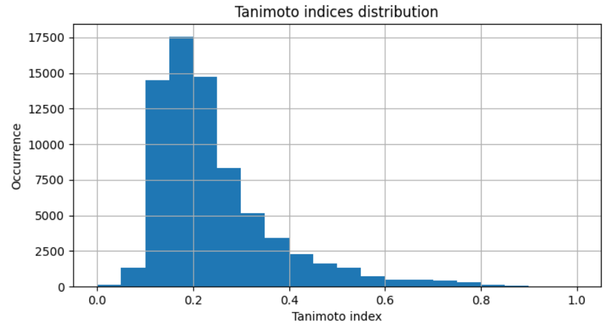

		CCR2	399	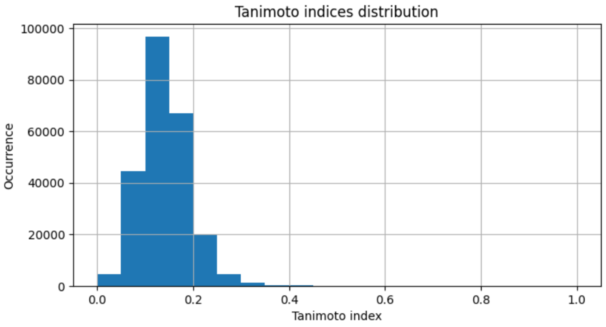
		CXCR3	199	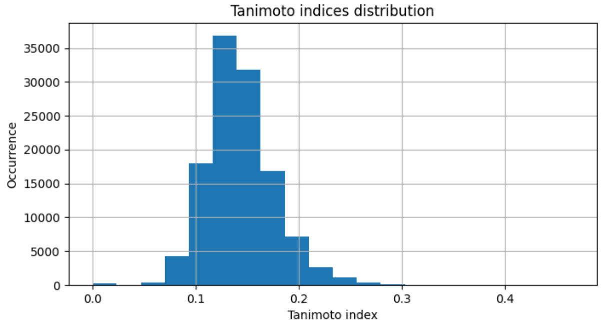
CXCR3	994	CXCR3	199	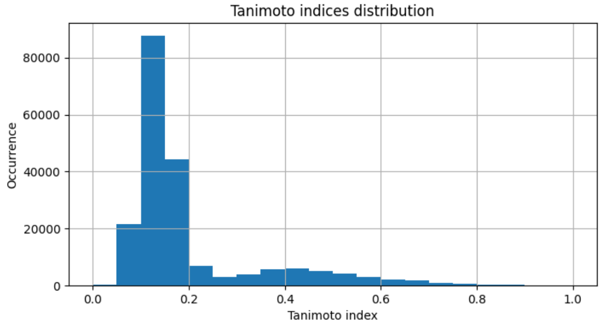
		CCR2	399	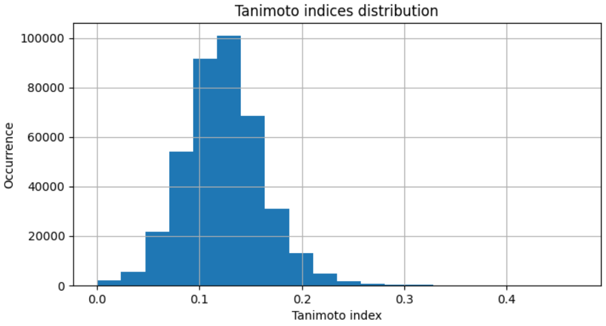

		CCR3	121	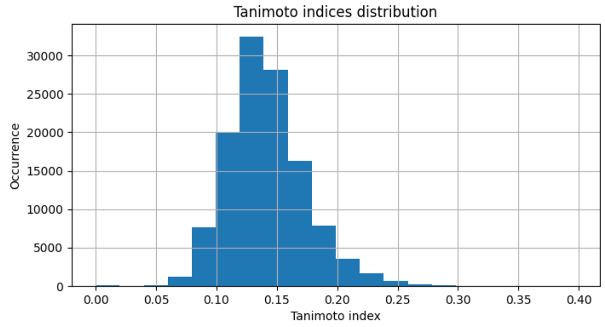
CB1	1566	CB1	314	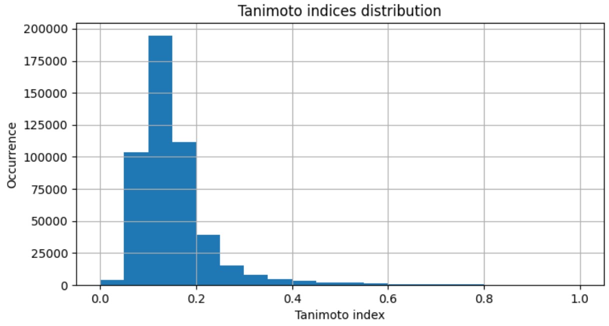
		CB2	418	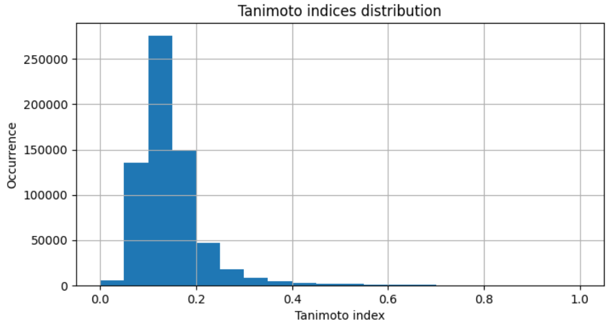
		CB2 selective	35	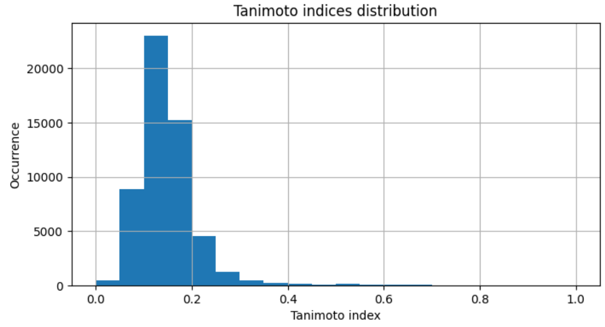

CB2	2093	CB2	418	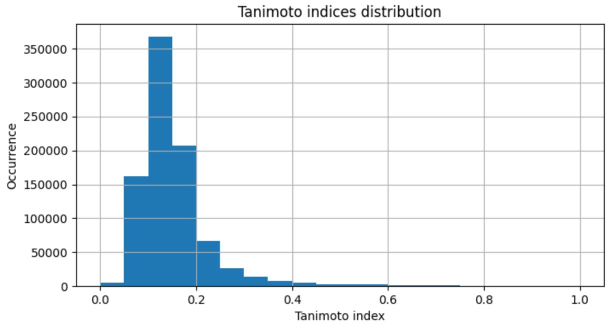
		CB1	314	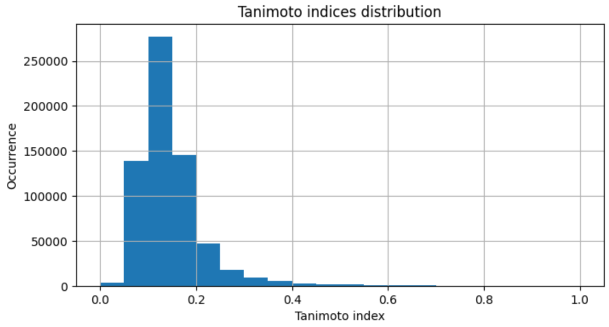
		CB2 selective	35	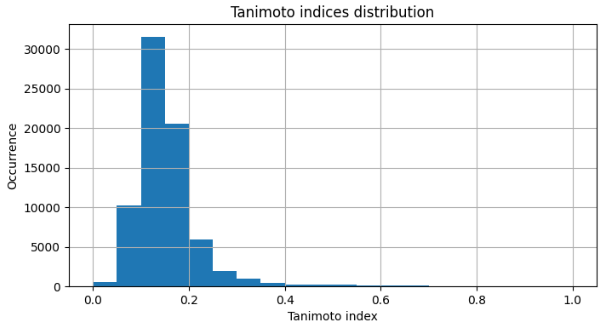

Table S10. Histograms of values of Tanimoto index between the previous [4,5] and the current training datasets retrieved from ChEMBL.

Previous training sets from https://db-gpcr.chem.uw.edu.pl	Number of datapoints	Current training sets including non-active compounds	Number of datapoints	Histogram of values of Tanimoto index<br><br>Previous vs. current dataset
CB1	1566	CB1	4509	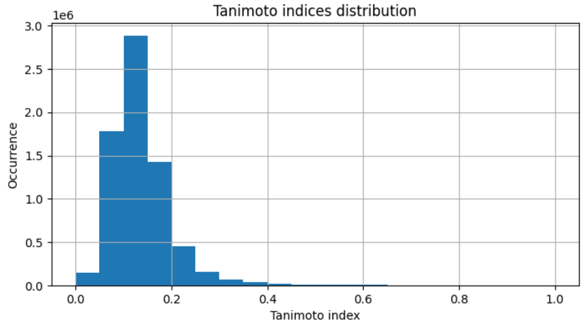
CB2	2093	CB2	4087	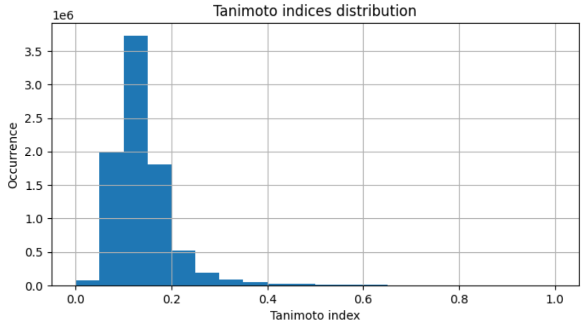

References

1. Ballesteros, J.A.; Weinstein, H. Integrated Methods for the Construction of Three-Dimensional Models and Computational Probing of Structure-Function Relations in G Protein-Coupled Receptors. *Methods in Neurosciences* **1995**, *25*, 366–428, doi:10.1016/S1043-9471(05)80049-7.
2. Madeira, F.; Pearce, M.; Tivey, A.R.N.; Basutkar, P.; Lee, J.; Edbali, O.; Madhusoodanan, N.; Kolesnikov, A.; Lopez, R. Search and Sequence Analysis Tools Services from EMBL-EBI in 2022. *Nucleic Acids Res* **2022**, *50*, W276–W279, doi:10.1093/nar/gkac240.
3. Maestro, Schrödinger Release 2022-3.
4. Mizera, M.; Latek, D. Ligand-Receptor Interactions and Machine Learning in GCGR and GLP-1R Drug Discovery. *IJMS* **2021**, *22*, 4060, doi:10.3390/ijms22084060.
5. Mizera, M.; Latek, D.; Cielecka-Piontek, J. Virtual Screening of C. Sativa Constituents for the Identification of Selective Ligands for Cannabinoid Receptor 2. *International Journal of Molecular Sciences* **2020**, *21*, 5308, doi:10.3390/ijms21155308.
